# Maltreatment-Associated Psychiatric Problems: An Example of Environmentally Triggered ESSENCE?

**DOI:** 10.1155/2013/148468

**Published:** 2013-04-17

**Authors:** Helen Minnis

**Affiliations:** Institute of Health and Wellbeing, University of Glasgow, Caledonia House, Glasgow G3 8SJ, UK

## Abstract

This paper presents a new concept—maltreatment associated psychiatric problems (MAPP)—a syndrome of overlapping complex neurodevelopmental problems in children who have experienced abuse or neglect in early life. Children with MAPP are a hidden population in the community and, in clinical settings, their problems can seem overwhelming. Individual disorders associated with maltreatment are discussed as well as the overlap between these disorders and their shared environmental and genetic predisposing factors. Because of the complex and overlapping nature of MAPP, with symptoms emerging in early life, I argue that it should be considered an example of ESSENCE. Children presenting with likely MAPP should receive a comprehensive assessment, probing for symptoms of all of the ESSENCE disorders and leading to the use of evidence-based treatments where these are available.

## 1. Maltreatment and Mental Health

Maltreated children are at significantly greater risk of developing psychiatric disorders compared to the general population [1] and these can have profoundly negative consequences across the lifespan: as a group and compared with the general population, maltreated children have significantly poorer educational [2] and employment [3] outcomes, are more likely to become homeless [4] or go to prison [5], and have poorer relationship and family functioning once they reach adulthood [6].

Much of the existing research literature has come from studies of children reared in institutions and there is now no doubt that emotional neglect in early life places children at risk of a wide range of problems including reactive attachment disorder (RAD) (characterised by indiscriminate friendliness and/or hypervigilance and emotional withdrawal); attention-deficit hyperactivity disorder (ADHD) (characterised by poor concentration, impulsivity, and overactivity); posttraumatic stress disorder (characterised by flashbacks, nightmares, and avoidance of reminders of traumatic events); depression and anxiety [7–9]. Similar findings have emerged from longitudinal studies of children maltreated within a family context [10]. Coupled with these psychiatric problems, maltreated children may also have cognitive problems [11], language problems [12] and motor problems [1]. 

## 2. What Are the Maltreatment-Associated Psychiatric Problems?

### 2.1. Reactive Attachment Disorder

The only disorder in the psychiatric classifications systems that is specifically associated with maltreatment is reactive attachment disorder (RAD). RAD is a serious disorder of social functioning associated with maltreatment with two subtypes: inhibited (wary, watchful behaviour) and disinhibited (overfriendly behaviour). The disinhibited form is known to be associated with significant psychiatric morbidity [13] and can persist despite changes in caregiving context [14]. Inhibited RAD is thought to be very rare beyond the specific context of maltreatment [14] but we have recently shown that the disinhibited form is far from being rare and has a prevalence of around 1.4% in a deprived population [15]—similar to or even higher than the population prevalence of ASD [16]. We have also found that children with RAD often have complex neurodevelopmental problems [17] and that, even after living for several years in loving adoptive families, these children can still have problems that are a major burden for themselves, their families, and their peers [18].

### 2.2. Attention-Deficit Hyperactivity Disorder (ADHD)

Although ADHD is fairly common in the general population with prevalence estimates of around 6-7% in children and 5% in adults [19], it is even more prevalent in maltreated [20] or postinstitutionalised populations [7]. Adoption in stable nurturing families does not necessarily ameliorate the problems: the rates of ADHD medication are almost 5 times higher for boys who were adopted internationally in Sweden compared to the general population [21]. This is something of a conundrum because ADHD is one of the best-researched disorders when it comes to genetics and is known to have a high heritability in the general population of around 70–90% [22]. In a recent review, however, Thapar and colleagues emphasise the close associations between genetic and environmental factors (e.g., a genetic predisposition in the mother to smoking may confound the environmental effects of smoking in the antenatal period on the development of ADHD) and suggest that, despite the strong evidence for heritability in ADHD, severe early adversity/privation has a strong enough evidence base to be considered a causal risk factor for ADHD [22]. 

### 2.3. Posttraumatic Stress Disorder

Unsurprisingly, posttraumatic stress disorder (PTSD) is common in populations of children who have been maltreated [23]. It is, however, less clear whether PTSD is distinct from inhibited reactive attachment disorder and the complexity of the presentation of trauma symptoms in abused and neglected children has prompted some to suggest that the term developmental trauma disorder may be more appropriate [24].

### 2.4. Conduct Disorder and Oppositional-Defiant Disorder [25]

It is well known that harsh parenting and early maltreatment are strongly associated with conduct disorder [26] but a history of maltreatment is also present in nearly half of children presenting to clinics with oppositional defiant disorder (ODD) [25]. Interestingly, children who were reared in Romanian orphanages and adopted to the UK do not appear to be at a higher risk of conduct disorder and ODD compared to family-reared comparison children [8], therefore there appears to be some—so far poorly understood—specificity as regards particular types of early adversity and particular developmental outcomes.

### 2.5. Anxiety and Depression

There is some debate as to whether or not anxiety and depression are particularly associated with maltreatment. Retrospective clinic-based studies have suggested that there is evidence of an association between childhood physical and sexual abuse and later anxiety disorders [27] and in the Bucharest Early Intervention Study, children who grew up in institutions were almost three times as likely to be diagnosed with an anxiety disorder at followup compared to children who grew up in families [7]. However, children adopted from Romania to the UK were not more likely, at age 6, to have anxiety or depression [8]. Interestingly, however, by adolescence higher than expected rates of anxiety and depression had emerged in the sample of Romanian children adopted to the UK and risk was influenced by both genetic factors (5HTT genotype) and environmental factors (stress during adolescence) [28]. 

## 3. What Is the Overlap?

In a detailed diagnostic study of 165 consecutive outpatients, Ford and colleagues found that where a diagnosis of comorbid ADHD/ODD was made, 73% of these children had a history of maltreatment compared to 26% of those with a diagnosis of ADHD alone and 48% of those with a diagnosis of ODD alone [25]. This has led us to suspect that comorbidity may be the rule, rather than the exception, in MAPP. In our own clinical research group, we have been struck by the neurodevelopmental complexity of the problems displayed by the subgroup of maltreated children who have psychiatric disorders. For example, in a study of adopted maltreated children with indiscriminate friendliness, there was a very high degree of psychiatric comorbidity with many children suffering from reactive attachment disorder, ADHD, and conduct and anxiety disorders, despite their having spent an average of 4 years with their adoptive parents [18]. This was not simply a feature of a selected clinical population: in a total population sample of around 1600 children, all children with a diagnosis of RAD also had other diagnoses including over half also suffering from ADHD and nearly a third suffering from conduct disorder [15]. 

## 4. Why Do Trajectories Differ So Much?

One of the great conundrums in the maltreatment field is why some children develop complex neurodevelopmental problems in the context of abuse and neglect yet others do not, despite having apparently suffered the same degree of early adversity. In the seminal studies by Rutter and colleagues, in which they followed up a random sample of the children adopted from Ceausescu's Romanian orphanages, despite more than two years of exposure to extreme neglect, between a fifth and a quarter were “free of any measurable dysfunction” at age 6 [8]. This perhaps implies that there are protective genetic factors that render some children resilient even to very adverse environmental circumstances. We showed, in a general population twin study, that despite being apparently caused by maltreatment, there is a substantial heritable component to the aetiology of RAD [29]. Various other groups have investigated the molecular basis of the “differential susceptibility hypothesis”—in which heritable characteristics render some children more at risk of developing psychiatric disorder in the context of maltreatment than others. For example, meta-analysis of several studies has now shown that the association between maltreatment and mental health problems is significantly stronger in the group of boys who have a genotype conferring low versus high monoamine oxidase A activity [29, 30], and a polymorphism at the dopamine D4 (DRD4) receptor renders some children at significantly higher risk of developing disruptive behavioural disorders in the context of insensitive parenting than others [31]. The molecular genetic research, however, also suggests a logic in regarding maltreatment-associated problems as overlapping ESSENCE disorders: for example, Thapar stated in her review of the aetiology of ADHD that “the genetic risks implicated in ADHD generally tend to have small effect sizes or be rare and often increase risk of many other types of psychopathology.” Little is known about *how* genes interact with the environment to produce differing outcomes, but one area of intense research scrutiny is the hypothalamic-pituitary axis. We have conducted a recent systematic review of stress responses in the context of maltreatment that demonstrated a wide range of effects on the stress response system of maltreatment depending on the sample [32] and differences in a child's ability to regulate stress hormones in response to adversity may potentiate the effects of maltreatment on development. 

## 5. Is There a Specific MAPP Phenotype?

Various groups, including our own, have found that the subgroup of maltreated children who have psychiatric problems tend to have overlapping complex difficulties that are often associated with language and other cognitive problems [7, 8, 18]. In addition, there tends to be a core problem with forming and maintaining close intimate relationships. This is undoubtedly true of children who have RAD, but a recent systematic review conducted by our group showed that problems with facial emotion recognition were common in a wide range of child psychiatric disorders [33]. Such difficulties in recognising the basic emotions crucial to successful social interaction are likely to be disabling for children with MAPP. A recent review of the literature on the brain effects of early maltreatment has suggested that a dysfunction in limbic brain circuits might impede the child's ability to respond to emotional faces appropriately leading to a particularly disabling mixture of attentional problems and problems with relationships [34].

## 6. What Are the Long-Term Outcomes of MAPP?

There are many unanswered questions about how MAPP might develop across the lifecourse but we do know that early adversity is associated with both physical and mental health problems [35, 36]. Maltreatment is now recognised as an important risk factor for both personality disorder [37] and schizophrenia [38] but we do not know what proportion of children with MAPP are at risk of these adult psychiatric disorders nor the extent to which MAPP is a necessary mediator between maltreatment and adult mental health problems. There is, however, a recognised developmental route from early harsh parenting through conduct disorder and on to various later health problems; for example, delinquent adolescents have 9 times the *all-cause* mortality compared to their nondelinquent peers [39]. The longitudinal course of MAPP will be an important focus for future study.

## 7. A Hidden Population

The one aspect of MAPP that mitigates against it being described as an ESSENCE group of problems is that of referral to services. A core feature of the ESSENCE concept is that these are early symptomatic syndromes recognised by parents and/or professionals that result in referral to services and some sort of neurodevelopmental clinical examination. Sadly, for children who are experiencing neglect, their problems may be neither recognised nor resulting in referral to services. An additional problem may be that, even when referred, the complexity of their difficulties is so overwhelming that they defy description by parents and paralyse clinicians. Our recent study of adopted children with complex neurodevelopmental problems [18] lends some support to this notion: despite very burdensome problems, many of which have evidence-based treatments available, only a small proportion of children were in touch with child and adolescent mental health services (CAMHS). Those parents who had had contact with CAMHS sometimes reported tense and frustrating interactions with clinicians who did not seem to know where to start in teasing apart the array of difficulties being presented to them. This group of children was, however, advantaged by living with assertive adoptive parents compared to children with RAD living in birth families. In our recent study of RAD prevalence in a deprived population, several home visits were sometimes required to achieve a face-to-face assessment of a child, even when parents had opted into the study [15]. This level of assertive outreach would be well beyond the capacity of CAMHS services as currently structured, certainly in the UK and probably in many other countries. This has led us to conclude that there may be a hidden population of children with complex neurodevelopmental problems being denied the interventions that could radically change their developmental trajectories for the better. Because this is potentially such an important group, whose problems need to be addressed as early as possible in order to avoid extremely negative outcomes, new models of assertive outreach and preventative and intervention services need to be developed if we are to reduce the burden to these children, their families, and to society as a whole.

## 8. Future Directions

A focus on children with MAPP as a group may allow more coherent and pragmatic longitudinal studies, ideally starting in the antenatal period, to follow this very vulnerable group closely in the early years and to track trajectories through life. More research needs to be conducted into how to identify these children early enough for preventative and therapeutic interventions to significantly alter trajectories for the better. It is very likely that existing evidence-based treatments, for example, pharmaceutical treatment for ADHD, will be effective in this group, but treatment trials will need to focus on recruiting and retaining children with MAPP if we are to be confident of this. There also needs to be a focus on developing preventative interventions and, again, this research will require very assertive techniques to recruit and retain samples. Key underexplored areas include the role of antenatal stress and toxins, parent-infant interaction in the early months and years of life, molecular genetics and epigenetics of MAPP as a whole and mechanisms at the level of brain structure and function. While the research evidence accrues regarding mechanisms, we must also be investigating new ways of reaching these children at the service level in order to deliver the services they need. Once engaged in services, a detailed diagnostic assessment covering all of the ESSENCE disorders is essential so that the right interventions can be put in place.
